# Integrating Diffusion Tensor Imaging and Neurite Orientation Dispersion and Density Imaging to Improve the Predictive Capabilities of CED Models

**DOI:** 10.1007/s10439-020-02598-7

**Published:** 2020-09-03

**Authors:** Marco Vidotto, Matteo Pederzani, Antonella Castellano, Valentina Pieri, Andrea Falini, Daniele Dini, Elena De Momi

**Affiliations:** 1 Department of Electronics, Information and Bioengineering, Politecnico di Milano, Milan, Italy; 2 Vita-Salute San Raffaele University, Milan, Italy; 3 Neuroradiology Unit and CERMAC, IRCCS San Raffaele Scientific Institute, Milan, Italy; 4 Department of Mechanical Engineering, Imperial College, London, UK

**Keywords:** Computational model, Hydraulic permeability, Drug delivery, NODDI, DTI

## Abstract

This paper aims to develop a comprehensive and subject-specific model to predict the drug reach in Convection-Enhanced Delivery (CED) interventions. To this end, we make use of an advance diffusion imaging technique, namely the Neurite Orientation Dispersion and Density Imaging (NODDI), to incorporate a more precise description of the brain microstructure into predictive computational models. The NODDI dataset is used to obtain a voxel-based quantification of the extracellular space volume fraction that we relate to the white matter (WM) permeability. Since the WM can be considered as a transversally isotropic porous medium, two equations, respectively for permeability parallel and perpendicular to the axons, are derived from a numerical analysis on a simplified geometrical model that reproduces flow through fibre bundles. This is followed by the simulation of the injection of a drug in a WM area of the brain and direct comparison of the outcomes of our results with a state-of-the-art model, which uses conventional diffusion tensor imaging. We demonstrate the relevance of the work by showing the impact of our newly derived permeability tensor on the predicted drug distribution, which differs significantly from the alternative model in terms of distribution shape, concentration profile and infusion linear penetration length.

## Introduction

The blood-brain barrier (BBB) is a highly selective semipermeable vascular system composed by endothelial cells, astrocyte end-feet, and pericytes that serves as a diffusion barrier.^3^ Despite the BBB is essential for the normal function of the central nervous system, it is also a dramatically effective barrier that prevents most drugs from going from the blood stream to the brain tissue.^3^ For this reason, the BBB has been clearly identified as the main cause of the failure of chemotherapeutic treatments that aim at targeting the brain tissue.^2^,^5^,^8^,^17^

To overcome this obstacle, an innovative and promising technique, namely convection-enhanced delivery (CED), has been introduced by Bobo *et al.*^5^ in 1994, consisting in injecting a therapeutic agent under positive pressure directly into the brain parenchyma by means of one or more catheters. CED was originally designed for treating patients with aggressive tumours, such as glioblastoma multiforme (GBM), where the survival rate is dramatically low. Indeed, only about 40% of them live more than a year after diagnosis.^30^ CED could offer a viable alternative to more conventional treatments, which consist in surgical resection followed by simultaneous radiation therapy and chemotherapy.^24^ Indeed, despite these treatment approaches being highly aggressive, patient outcomes remain dismal and around 80% of them experience tumoral recurrence or progression in the following years.^8^,^10^ Moreover, CED has been recently used for delivering therapeutic substances for other brain diseases such as gene therapy for Parkinson’s disease^7^ and antiseizure agents for epilepsy.^33^

Regardless of the pathology, a pivotal factor for CED efficacy is the ability to reach all the diseased tissue with enough concentration of therapeutic agent, and, on the other hand, to leave the healthy tissue unaltered as much as possible to avoid side effects.^8^,^11^,^17^ To optimise the treatment and to obtain better clinical outcomes, a valuable support is given by numerical models.^40^ In fact, by modelling the brain structures and the drug characteristics, they can predict how the drug will distribute in the brain for a given initial catheter setup. In this way, in the preoperative phase, the surgeon can examine different clinical settings (e.g. catheter placement, infusion flow rate) and plan the best way to proceed.^8^,^17^

Nevertheless, since brain is an anisotropic and heterogeneous porous tissue composed of grey matter (GM), white matter (WM), cerebrospinal fluid (CSF) and blood vessels (BV), modelling CED is extremely challenging.^16^,^40^ In the last years, several researchers have proposed numerical models based on different hypotheses and assumptions.^12^,^20^,^23^,^31^,^34^,^35^,^43^ However, their predictions do not always match the experimental observations, thus suggesting that there is still a long way to go to guarantee an accurate targeting of the zones of interest. This can be due to several reasons, but a key factor is related to how the microstructure of the brain tissue is modelled, both at the injection site and in the targeted area.^41^

The previously mentioned investigations used diffusion tensor imaging (DTI) to characterize, *non-invasively,* the tissue microstructure which in turn affects the penetration of the drug molecules. Indeed, DTI is a *non-invasive* imaging modality that measures the effects on the magnetic resonance signal intensity of water molecules diffusion over time. The resulting diffusivity tensor is isotropic in GM areas, mainly composed by cell bodies, and anisotropic in WM areas, whose microstructure is dominated by axonal fibres.^29^ DTI represents a powerful tool because it allows inferring the orientation of the neural fibres and distinguishing between GM and WM. This provides important information that can be used to define specific modelling parameters, such as, the eigenvectors of the permeability and diffusivity tensors. Despite DTI being extremely useful, it has an important limitation in that it does not allow obtaining any information about the extracellular space volume fraction (${\text{VF}}_{\text{ECS}}$) of the brain tissue. Critical modelling parameters, such as hydraulic permeability, are directly related to the ${\text{VF}}_{\text{ECS}}$^41^; therefore, using DTI in isolation to derive inputs for modelling purposes has an important drawback, in turn affecting the currently available models.

In this work, we aim at overcoming the limitations of the DTI-based approach and improving the model predictive capability by discussing a methodology that enables to consider the information related to the ${\text{VF}}_{\text{ECS}}$ and we demonstrate its use in a single case-study of a healthy subject. In contrast to previous models, we not only consider DTI-derived information, but we also incorporate a more comprehensive quantification of the brain tissue microstructural complexity derived from a more sophisticated multicompartmental diffusion model, namely the neurite orientation dispersion and density Imaging (NODDI).^44^ Indeed, NODDI relies on more complex diffusion data fitted by a tissue model that distinguishes three compartments with different microstructural characteristics: intracellular, extracellular and CSF compartments. Each environment affects the diffusion of the molecules differently, thus giving rise to separate signals. By estimating the relative contribution of the three distinct compartments to the total diffusion signal in each voxel, it is possible to infer the ${\text{VF}}_{\text{ECS}}$ in every part of the brain. First, we derive a relationship between ${\text{VF}}_{\text{ECS}}$ and WM hydraulic permeability from the numerical analysis of a simplified geometrical model. We then demonstrate the relevance of the work by comparing our model with another state-of-the-art model by conducting the same drug delivery simulations and comparing the outputs in terms of infusion volume and shape. We show that the predictions given by our model differ significantly from those given by the models that use only DTI and using a fixed value of permeability. We finally discuss the important implications that the new CED modelling framework has in terms of its potential future use in pre-clinical trials.

## Materials and Methods

### Imaging Dataset

The brain is a porous medium, where the solid part is composed of neurons and glial cells, and the voids represent the extracellular space.^27^ Accordingly, information relative to the volume fraction occupied by each region is important to describe brain properties. With NODDI, it is possible to extract, for each voxel, the volume fraction occupied by the intraneurite (${\text{VF}}_{\text{INC}}$) and isotropic (${\text{VF}}_{\text{Water}}$) compartments. Each environment affects the diffusion of the molecules with different contributions giving rise to separate signals.^44^ In this work, we used an imaging dataset acquired on a healthy adult subject on a 3T Ingenia CX scanner (Philips Healthcare, Best, The Netherlands), with a 32-channel head coil. The study was approved by the OSR Institutional Ethics Committee, and signed informed consent was provided by the subject before magnetic resonance imaging (MRI).

The MRI protocol included:

- a two-shell diffusion MRI (DMRI) sequence, based on axial Single-Shot Spin-Echo echo planar imaging (EPI). Diffusion gradients were applied along 35 and 60 non-collinear directions, and images were acquired at multiple *b*-values (0, 711, and 3000 s/mm^2^), with the following parameters: TR/TE 5977/78 ms; flip angle, 90°; 60 slices; thickness, 2/0 mm gap; acquisition matrix, 128 × 126; voxel size, 2 × 2 × 2 mm; SENSitivity-Encoding (SENSE) reduction factor, *R* = 2; Multiband factor = 2. Twelve images without diffusion weighting (*b* = 0 s/mm^2^) were obtained, one of which was acquired with reversed phase-encoding to estimate susceptibility-induced distortions. This diffusion imaging dataset was exploited to extract both tensorial and NODDI metrics, to be combined in the model.
- a sagittal 3DT1-weighted sequence, acquired with the following parameters: repetition time/echo time [TR/TE] 12/5.9 ms; flip angle, 8°; 236 slices; thickness, 0.8/0 mm gap; acquisition matrix, 320 × 299; voxel size, 0.8 × 0.8 × 0.8 mm; SENSE factor, *R* = 2; acquisition time, 5 min 19 s. This anatomical sequence was exploited to achieve the preoperative planning of the simulated gadolinium (GD) solution infusions along nine different catheter orientations.

DMRI volumes were corrected for eddy-current distortions, movement and susceptibility-induced artifacts by applying the ‘*eddy*’ and ‘*top-up*’ tools of FMRIB Software Library, respectively (FSL, University of Oxford, https://fsl.fmrib.ox.ac.uk/fsl/).

DTI analysis was performed on high angular resolution diffusion-weighted Imaging (HARDI) volumes (60 diffusion directions, *b*-value = 3000 s/mm^2^) extracted from the multi b-value DMRI dataset using the ‘*fslsplit*’ and ‘*fslmerge*’ FSL-tools. The *‘dtifit’* FSL*-*tool allowed estimating the diffusion tensor and generating tensorial maps, such as Fractional Anisotropy (FA) which measures the fraction of the diffusion that is anisotropic.^29^

The NODDI model was fitted to all the volumes of the two-shell DMRI datasets using the MATLAB NODDI toolbox (http://mig.cs.ucl.ac.uk/Tutorial.NODDImatlab), that computed the ${\text{VF}}_{\text{INC}}$ and ${\text{VF}}_{\text{Water}}$ diffusion compartments of each voxel. Those outputs were then reparameterized in order to derive the extraneurite diffusion compartment (${\text{VF}}_{\text{ENC}}$), so that the sum of the three compartments equaled 1 in each voxel, as described in Caverzasi *et al*.^6^ Hence, the ${\text{VF}}_{\text{ECS}}$ that we integrated in our model was finally derived as ${\text{VF}}_{\text{ENC}} + {\text{VF}}_{\text{Water}}$, which corresponds to the sum of the compartments where a drug can flow.^39^

### Brain Tissue Modelling

The brain is an extremely complex system whose tissue mainly consists of cells immersed in the CSF. Despite most of the relevant literature agrees to describe the brain as a porous medium, there is not a common answer on which specific model is the most appropriate to use. Indeed, one of the most controversial aspect is the modelling of the solid part that can be described as deformable or rigid. For example, some authors use linear elastic^35^ or hyperelastic^12^ material constitutive laws, whereas others model the fiber bundles and other soft solid components of the brain as not-deformable.^9^,^19^,^20^ Despite deformable models offer a more accurate description because they consider the infusion-induced tissue deformation, they also require a larger number of parameters, that could be hard to identify, and require a high computational cost. On the other hand, rigid models demonstrated to be reliable when the deformation can be neglected and with a much lower computational cost with respect to elastic models.

In this study, we have adopted a rigid approach (whereby the solid part of the tissue is considered undeformed throughout the simulations), that was used also by other authors,^9^,^19^,^20^,^41^ because we expect negligible tissue deformations to arise during the procedure identified for the comparison performed in this work. This assumption is supported by the findings of Garcìa *et al*.^14^ that, in a numerical study with comparable tissue properties, catheter dimension and flow rate, computed an average deformation about 0.02 in the immediate proximity of the catheter tip and below 0.009 in the vast majority of the remaining area. These results suggest that, for low injection rate, the deformation is not very significant and, most importantly, it is very localized in the proximity of the catheter tip. For these reasons, we modelled the brain tissue as a simpler and computationally less expensive rigid model whose continuity equation is^41^:

1 $$\nabla \cdot v = 0$$

where $v$ is the average extracellular fluid velocity and $\nabla$ is the gradient operator. Moreover, fluid flow in a porous medium is described by Darcy’s law:^20^

2 $$v = - \frac{K}{\mu } \cdot \nabla p$$

where $p$ is the fluid pressure, $\mu$ is the viscosity^41^ and $\varvec{K}$ is the hydraulic permeability tensor. Mass transport in the brain tissue is driven by convection, diffusion and loss due to absorption or washout:

3 $$\frac{\partial c}{\partial t} = - \nabla \cdot \left( {vc} \right) + \nabla \cdot \left( {\varvec{D} \cdot \nabla c} \right) - S$$

where *c* is the GD concentration, $S$ is the loss term ($0.01 \min^{ - 1}$)^26^ and ***D*** is the diffusivity tensor.

The brain model was divided in three areas following the thresholding suggested by Kim *et al.*,^20^ as summarized in Table 1. Despite a step transition between the different areas can induce a nonlinear behavior, currently available imaging modalities do not allow a more gradual passage. Moreover, in this study we limited the infusion area to the WM thus avoiding the aforementioned problem.

**Table 1:** Thresholding applied to divide the brain model between GM, WM and CSF.

Tissue region	Threshold range
Gray matter	$0 < {\text{FA}} < 0.23$
White matter	$0.23 < {\text{FA}} < 1$
Cerebro-spinal fluid	${\text{VF}}_{\text{Water}} > 0.99$

Each area has different properties that define the permeability and diffusivity tensors as it will be explained in the next sections.

### Diffusion Tensor

GM regions are characterized by isotropic diffusion. Accordingly, $\varvec{D}_{{\varvec{GM}}}$ is defined as:

4 $$\varvec{D}_{{\varvec{GM}}} = D_{0} \cdot \varvec{I}$$

where $D_{0}$ is the GD apparent molecular diffusivity equal to $1.54 \times 10^{ - 6} \,{\text{cm}}^{2} /{\text{s}}$, estimated from the empirical formula derived by Swabb *et al*.^36^,^43^ and $\varvec{I}$ is the (3 × 3) identity matrix.

On the contrary, in WM, diffusion is anisotropic. The principal direction of the diffusion tensor $\varvec{D}_{{\varvec{WM}}}$ was assumed as the maximum transport direction along the axons, as measured by DTI. Since water diffusion tensor $\varvec{D}$ is symmetric and positive definite, it is possible to define three orthogonal unit eigenvectors ($\overrightarrow {{e_{1} }}$, $\overrightarrow {{e_{2} }}$, $\overrightarrow {{e_{3} }}$) with the corresponding eigenvalues ($\lambda_{1}$, $\lambda_{2}$, $\lambda_{3}$). To account for the molecular transport of GD, eigenvalues obtained from $\varvec{D}$ at each voxel must be scaled according to the GD molecular diffusivity, as described in Linninger *et al.*,^23^ following these steps.

Step 1:

5 $$\varvec{D}E = E\varvec{\varLambda};\quad {\text{where}}\,\varvec{\varLambda}= \left[ {\begin{array}{*{20}c} {\lambda_{1} } & 0 & 0 \\ 0 & {\lambda_{2} } & 0 \\ 0 & 0 & {\lambda_{3} } \\ \end{array} } \right]\,{\text{and}}\,E = \left[ {\overrightarrow {{e_{1} }} ,\overrightarrow {{e_{2} }} ,\overrightarrow {{e_{3} }} } \right]$$

Step 2:

6 $$\varvec{D}_{{\varvec{WM}}} = \frac{{D_{0} }}{{\bar{\lambda }}} \cdot E\left[ {\begin{array}{*{20}c} {\lambda_{1} } & 0 & 0 \\ 0 & {\lambda_{2} } & 0 \\ 0 & 0 & {\lambda_{3} } \\ \end{array} } \right]E^{\text{T}} \quad {\text{where}}\;\bar{\lambda } = \frac{1}{3}\mathop \sum \limits_{i = 1}^{3} \lambda_{i}$$

### Permeability Tensor

Tensor $\varvec{K}$ was characterized differently in GM and WM areas. Since in GM, $\varvec{K}$ can be considered isotropic, $\varvec{K}_{{\varvec{GM}}}$ was defined by the following equation:

7 $$\varvec{K}_{{\varvec{GM}}} = K_{0} \cdot \varvec{I}$$

where $K_{0}$ is equal to $4.22 \times 10^{ - 18} {\text{m}}^{2}$.^20^

On the other hand, in WM areas, the permeability tensor $\varvec{K}_{{\varvec{WM}}}$ is transversely isotropic with the main transport direction that coincides with the one identified for $\varvec{D}_{{\varvec{WM}}}$.

8 $$\varvec{K}_{{\varvec{WM}}} = E\left[ {\begin{array}{*{20}c} {k_{\parallel } } & 0 & 0 \\ 0 & {k_{ \bot } } & 0 \\ 0 & 0 & {k_{ \bot } } \\ \end{array} } \right]E^{\text{T}}$$

where $k_{\parallel }$ and $k_{ \bot }$ describe the parallel and perpendicular hydraulic permeability with respect to the WM fibers. In previous studies,^19^,^20^ these parameters had a fixed values in all the brain ($k_{\parallel } = 6.75 \times 10^{ - 15} {\text{m}}^{2}$ and $k_{ \bot } = 4.22 \times 10^{ - 16} {\text{m}}^{2}$). On the contrary, in the proposed model, $k_{\parallel }$ and $k_{ \bot }$change spatially as a function of ${\text{VF}}_{\text{ECS}}$ as detailed in the next section. This represents the main element of novelty of the paper which introduces a new paradigm in defining the permeability tensor as a function of the WM microstructural organization.

#### Geometrical Model

**Figure 1:**
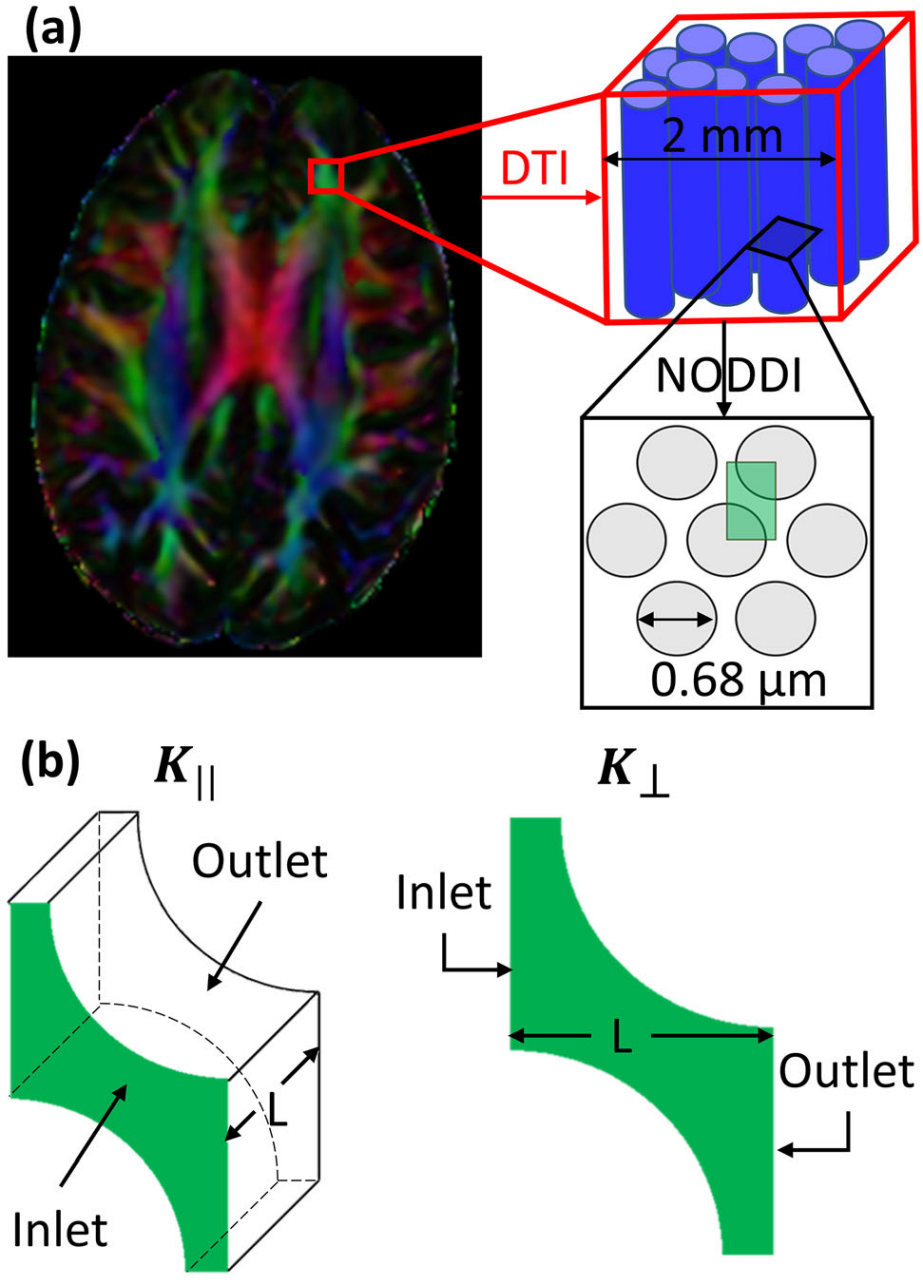
(a) This picture offers a schematic representation of the leading idea behind this study which combines both DTI and NODDI imaging modalities. DTI provides information about the WM fibres directionality: on the left, an axial section of FA map of a healthy subject, displayed as colour-orientation map. Latero-lateral-oriented fibres are coded in red, cranio-caudal fibres in blue, and antero-posterior fibres in green. The neural fibres orientation (red box) is used to define the permeability tensor eigenvectors whereas NODDI, providing an insight into the axonal microstructure (black box), allows deriving the permeability tensor eigenvalues. To do so, the WM is modelled as a triangular arrangement of fibres where each grey circle represents the section of an axon and the green box is the representative volume element (RVE) analysed.^41^ (b) Model geometries used to compute $k_{\parallel }$ and $k_{ \bot }$. The green shapes represent the extracellular space of each geometry, namely, the space where the fluid can flow, which has been measured being in tens of nanometres.^37^ On the left, 3D geometry used to simulate a flow parallel to the fibres with $L = 0.15 \mu {\text{m}}$. On the right, the bi-dimensional geometry used to simulate a flow perpendicular to the direction of the fibres with *L* that varied according to different values of ${\text{VF}}_{\text{ECS}}$.

To study how $k_{\parallel }$ and $k_{ \bot }$ are related to ${\text{VF}}_{\text{ECS}}$, we conducted a numerical analysis on a simplified model geometry resembling the WM structure. Adopting an approach similar to the one developed in Vidotto *et al.*,^41^ the axons were simulated as cylinders with rigid walls and the extracellular space corresponding to the space where the fluid could flow. Even in this case, as explained in the *Brain tissue modelling* section, it is possible to safely consider the axons as rigid if the flow rate is very low. The axons, with constant radius $r = 0.34 \mu {\text{m}}$,^22^ were organized following a triangular arrangement (Fig. 1a). Then, varying the distance between the axons, it was possible to obtain a set of geometries with ${\text{VF}}_{\text{ECS}}$ ranging from 0.15 to 0.80. Note that we performed more simulations in the interval between 0.15 and 0.40 because it is the physiological range indicated in Syková *et al.*^37^ Moreover, it is necessary to specify that describing the axonal bundles with arrays of constant-radius cylinders is an important assumption that will be argued in the *Discussion* section comparing our results with other studies.^13^,^41^

For each geometry, a pressure difference of 5 Pa was applied between inlet and outlet with no slip condition at the walls (Fig. 1b). This pressure was chosen to maintain a very low flow rate and to satisfy the assumptions that allow Darcy’s law to be used to model flow in the tissue. The average velocity within the medium, along the direction over which the gradient of pressure was applied, was computed solving the Navier-Stokes equations by means of the finite element method (FEM) solver ANSYS (ANSYS, Lebanon, NH) with semi-implicit methods for pressure linked equations. Pressure and velocity contours of two of the geometries used are reported in Appendix 2. Then, both $k_{\parallel }$ and $k_{ \bot }$ were computed using Darcy’s law (Eq. (2)). Finally, the numerical results describing the relation between ${\text{VF}}_{\text{ECS}}$ and permeability were fitted using the analytical equations developed by Tamayol and Bahrami^38^ for $k_{\parallel }$ and Kuwabara^21^ for $k_{ \bot }$. We selected these equations as they were shown to be the most accurate models in the comparative analysis on permeability of fiber bundles performed by Karaki *et al*.^18^ The resulting equations, whose coefficients were obtained using a generalized reduced gradient nonlinear solver, are reported below:

9 $$k_{\parallel } = \frac{{r^{2} }}{{2.97\left( {1 - {\text{VF}}_{\text{ECS}} } \right)}}\left( { - 1.47 - 0.94{ \ln }\left( {1 - {\text{VF}}_{\text{ECS}} } \right) + 2\left( {1 - {\text{VF}}_{\text{ECS}} } \right) - 0.5\left( {1 - {\text{VF}}_{\text{ECS}} } \right)^{2} - 0.039\left( {1 - {\text{VF}}_{\text{ECS}} } \right)^{4} } \right)$$

10 $$k_{ \bot } = \frac{{r^{2} }}{{7.77\left( {1 - {\text{VF}}_{\text{ECS}} } \right)}}\left( { - 1.56 - 1.04{ \ln }\left( {1 - {\text{VF}}_{\text{ECS}} } \right) + 2.05\left( {1 - {\text{VF}}_{\text{ECS}} } \right) - 0.5\left( {1 - {\text{VF}}_{\text{ECS}} } \right)^{2} } \right)$$

### CED Simulation Set-Up

The proposed methodology, where the permeability tensor eigenvalues are expressed as a function of the ${\text{VF}}_{\text{ECS}}$, was compared with a state-of-the-art DTI approach^9^,^19^,^20^ which assumes that $k_{\parallel }$ and $k_{ \bot }$ can be considered constant across all the WM areas. A critical point of the latter approach is that, as underlined by many studies,^12^,^35^,^41^ permeability is still a very controversial parameter with a wide range of values available in the literature. For brevity, we name our model the *DTI-NODDI model* and we will refer to the more conventional models, such as those developed by Kim *et al*.^19^,^20^ and Dai *et al.*,^9^ as the *DTI model*.

**Figure 2:**
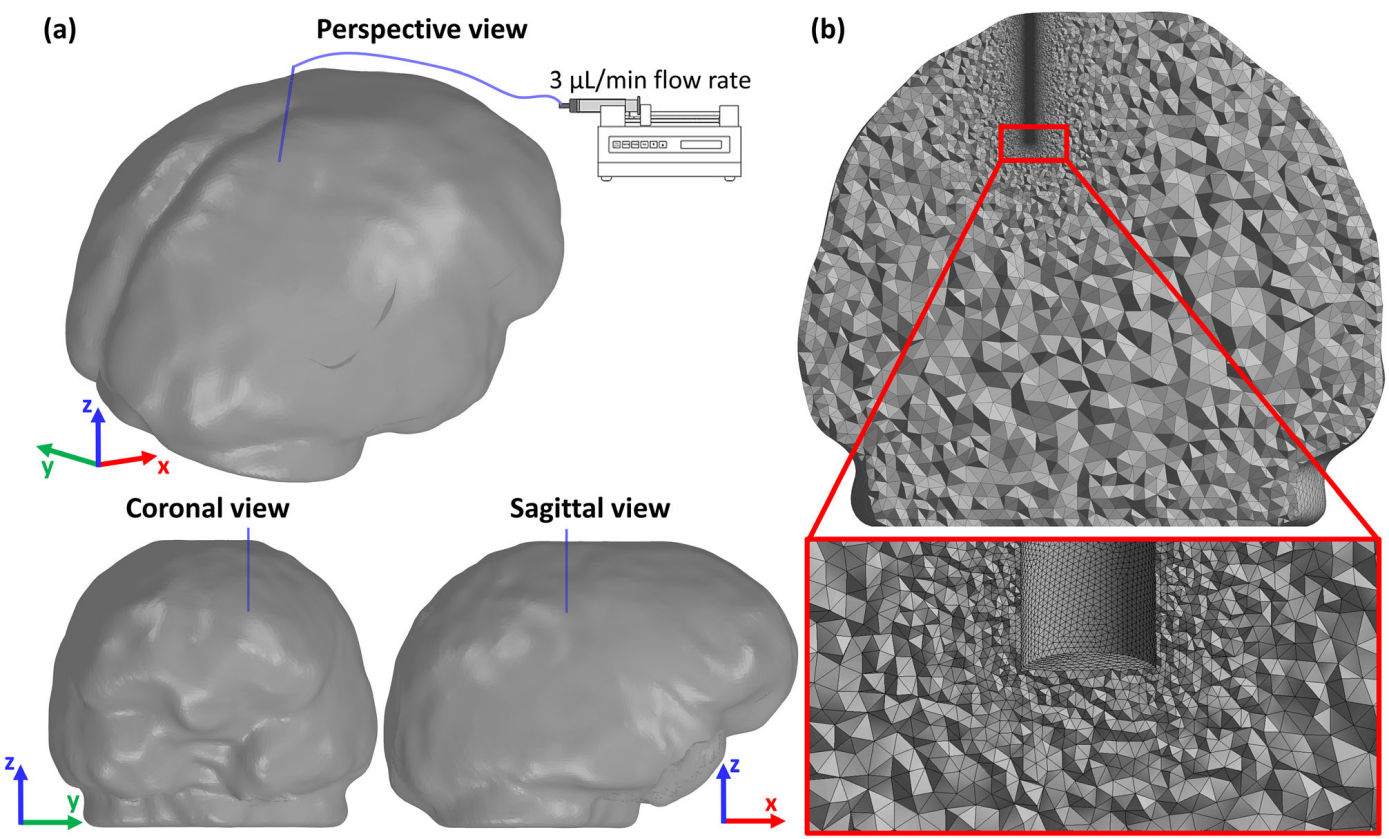
(a) Perspective view, coronal view and sagittal view of the brain model reconstructed from the healthy control DMRI dataset, with the infusion catheter inserted in a WM region. The model simulates a constant infusion rate of $3 \mu {\text{L}}/\hbox{min}$. (b) Coronal section plane with a detail of the final mesh adopted for all the simulation after sensitivity analysis (Appendix 1).

**Figure 3:**
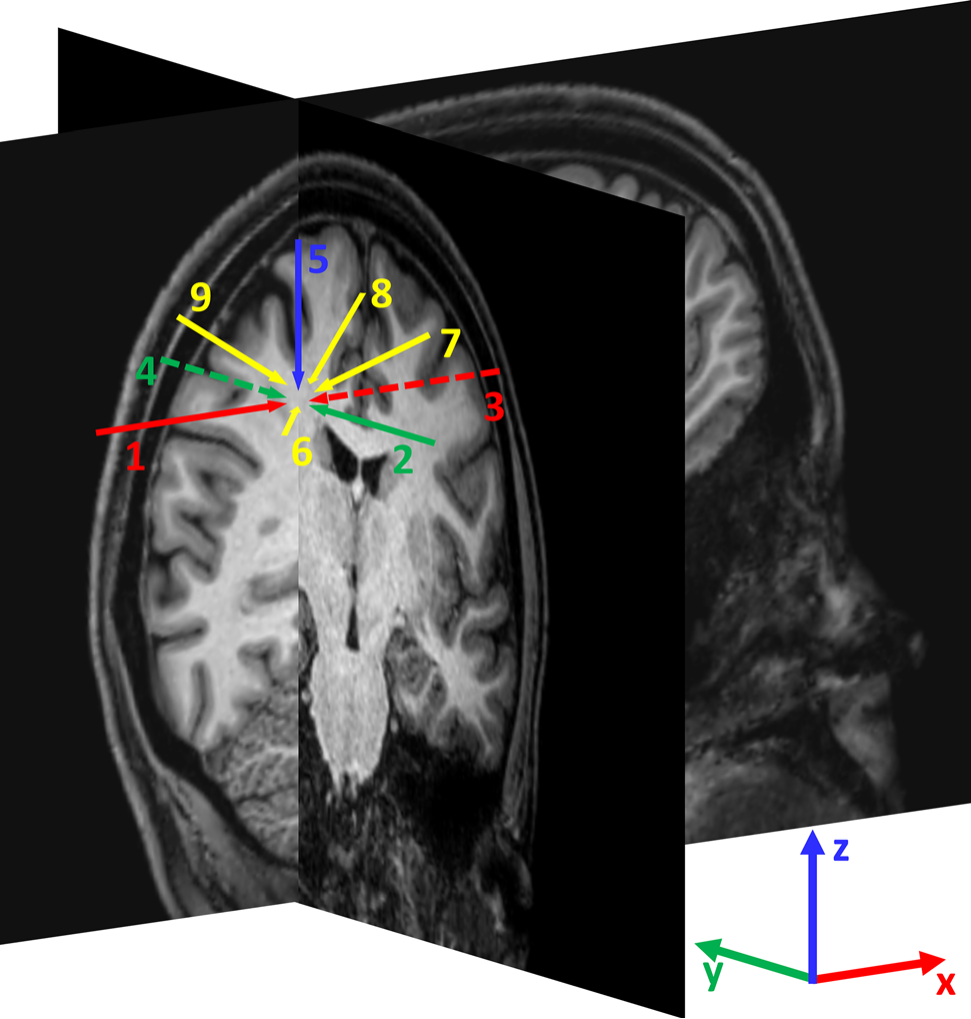
Scheme of the nine catheter orientations used to simulate the injection of the drug. To define the orientations, the brain coronal and sagittal planes intersecting the injection point are shown on 3D-T1 weighted sequence. Catheters 1 and 3 lie on the sagittal plane and in parallel with the *x* axis; catheters 2 and 4 lie on the coronal plane in parallel with the *y* axis; catheter 5 lie on the intersection between the sagittal and coronal planes; catheters 6, 7, 8, and 9 lie on the bisector of the solid angle defined by the semi-axes [-x, -y, z], [x, -y, z], [x, y, z] and [-x, y, z] respectively.

Both models simulate an injection of a GD solution in a WM area by means of a 1 mm diameter catheter (Fig. 2a). As boundary conditions for the flow, a uniform velocity profile was set at the inlet whose direction was aligned to that of the catheter. The simulations were then performed using a fixed infusion rate of $3 \mu {\text{L}}/\hbox{min}$,^4^ and zero pressure at the brain outer surface. Moreover, GD concentration at the inlet was fixed to $c_{0} = 0.5 {\text{mol}}/{\text{L}}$, and no solute flux was allowed outside the brain because of the presence of the *glia limitans*. The latter is a thin barrier of astrocyte foot processes surrounding the brain that acts as a physical barrier that insulates the parenchyma from unwanted molecules, thus making drug migration very difficult.^43^ The entire volume was discretized with about $3.5 \times 10^{6}$ tetrahedral elements after performing a mesh sensitivity analysis (Appendix 1). Note that a finer mesh was used in proximity of the catheter (Fig. 2b). The total infusion time, equal to 180 s, was chosen empirically as a convenient time frame to maintain the infused volume confined in a WM area. All simulations were systematically checked and numerical solutions converged to a stable steady state. The simulations were repeated for nine different orientations of the catheter with the solver ANSYS (ANSYS, Lebanon, NH) (Fig. 3).

### Metrics

Quantitative analyses were performed to examine the difference between the DTI-NODDI model and the DTI model in terms of drug concentration and distribution.

#### Concentration

An analysis of the drug distribution was carried out to investigate differences in the prediction of drug concentration in the tissue. The root mean square difference (RMSD) represents a good parameter to evaluate how much the results predicted by the models differ in terms of concentration.^31^ It is defined as:

11 $${\text{RMSD}} = \frac{1}{{c_{0} }}\sqrt {\frac{1}{{V_{\text{DTI}} \cup V_{\text{DTI - NODDI}} }}\mathop \sum \limits_{i = 1}^{n} V_{i} \left( {c_{\text{DTI}} - c_{\text{DTI - NODDI}} } \right)^{2} }$$

where $V_{\text{DTI}}$ and $V_{\text{DTI - NODDI}}$ are the models infusion volume where, similarly to Raghavan *et al.*,^31^ the drug concentration is higher than 2.5% of the infused concentration ($c_{ \hbox{min} }$); $V_{i}$ is the volume of each element of the mesh belonging to $V_{\text{DTI}} \cup V_{\text{DTI - NODDI}}$ and $c_{\text{DTI}}$ and $c_{\text{DTI - NODDI}}$ are the local concentrations predicted by the DTI and DTI-NODDI model, respectively.

#### Main Distribution Direction

The main direction along which the drug tends to distribute in the brain tissue was analyzed by means of the principal component analysis (PCA).^1^ The PCA was conducted on the coordinates of each element where the presence of drug was detected. Even in this case, we considered only the voxels with a concentration higher than $c_{ \hbox{min} }$. Performing a PCA on these data returns the principal direction along which the drug spreading has occurred.

From the PCA output, two additional analyses were conducted. In the first, we computed the angular difference between the infusion volumes principal directions in the two models at 180 s ($\vartheta$).

In the second, we compared the linear penetration length ($L_{ \hbox{max} }$) of the infusion volumes computed along the principal direction defined by the PCA.

## Results

### Permeability Tensor Characterization

**Figure 4:**
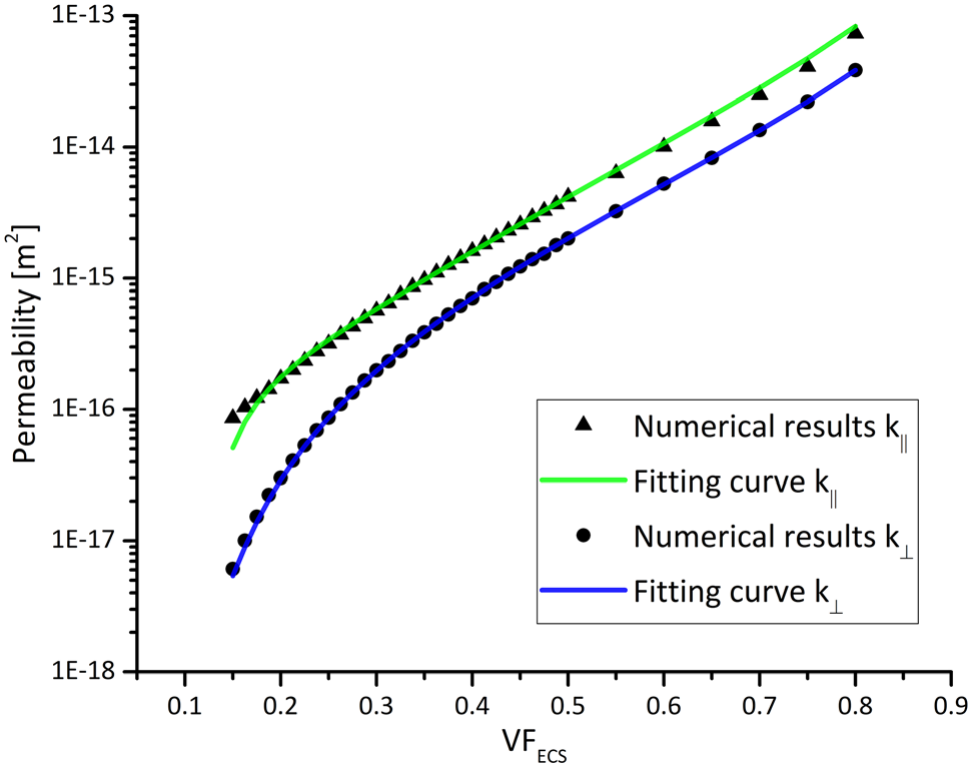
The parallel and perpendicular permeability returned by Eqs. (9) and (10) after fitting the numerical results are plotted vs. the ${\text{VF}}_{\text{ECS}}$.

The results of the numerical study to characterize the permeability tensor are graphically shown in Fig. 4. The numerical permeability values were fitted finding the coefficients of the analytical equations developed in Tamayol and Bahrami^38^ and Kuwabara^21^ for $k_{\parallel }$ and $k_{ \bot }$, respectively. Both parallel (Eq. (9)) and perpendicular (Eq. (10)) permeabilities have a similar trend which grows logarithmically as ${\text{VF}}_{\text{ECS}}$ increases.

### GD Concentration Distribution

**Figure 5:**
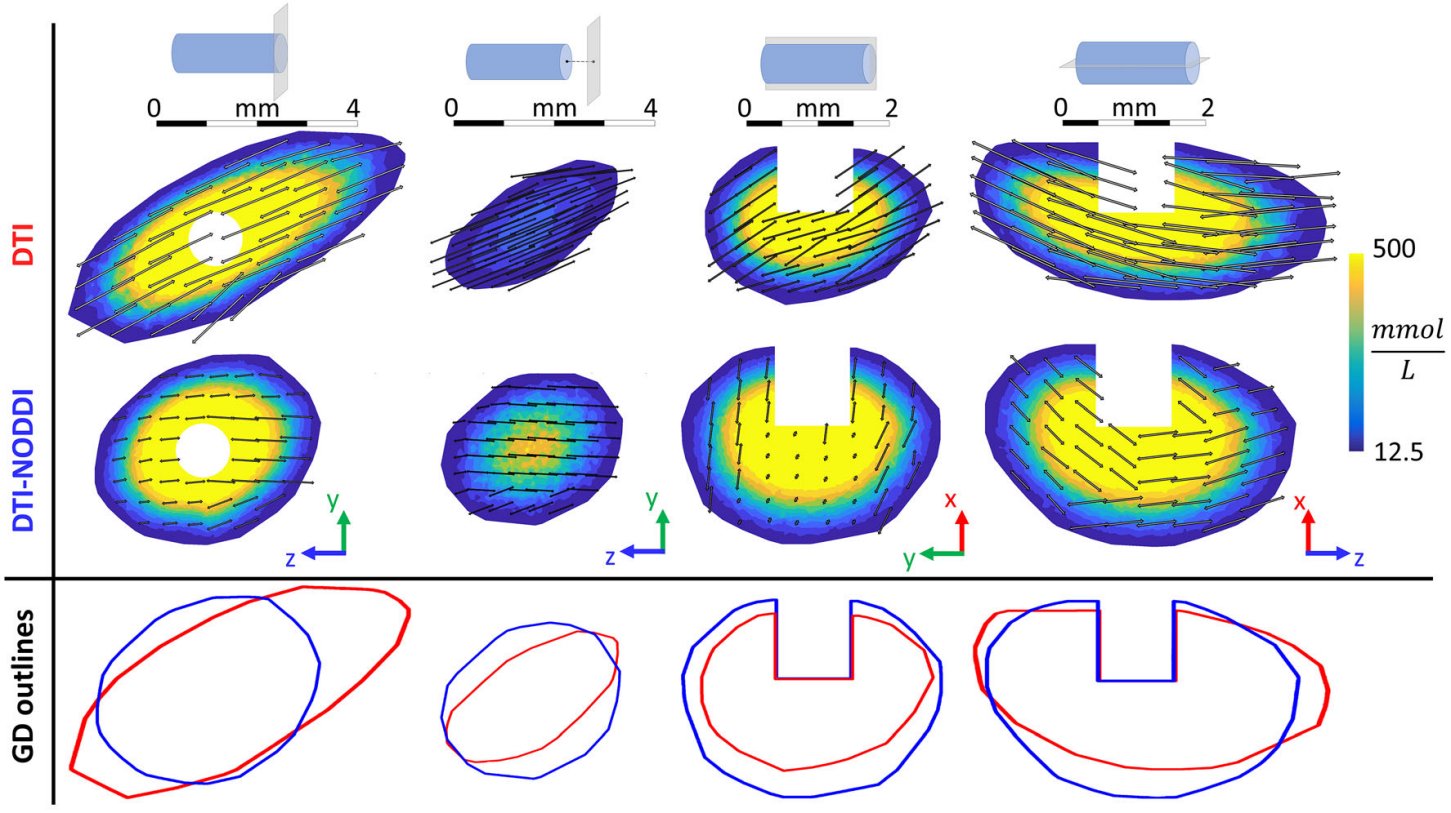
Predicted GD concentration after infusion in a WM region of the brain. Top: schematic drawing representing the catheter and the section plane corresponding to the contours below. Middle: GD concentration contours obtained with the DTI and the DTI-NODDI models at 180 s. The double headed arrows represent the resulting permeability vectors on different voxels. They were obtained by summing the parallel and perpendicular components of the permeability tensor and then projecting the resulting vector on the relevant plane. Bottom: Comparison between the DTI model (red) and the DTI-NODDI model (blue) in terms of GD distribution outlines defined as the more external elements with a GD concentration higher than $c_{ \hbox{min} }$.

Figure 5 shows an example of the predicted GD concentration after infusion in a WM region for both models on different section planes. From a qualitative point of view both differences in shape and in concentration distribution can be noticed from the contour plots. In particular, a marked difference in the shape of the areas is evident comparing the GD distribution outlines obtained with the two models (Fig. 5 bottom row). The outlines were defined finding the more external elements with a GD concentration higher than $c_{ \hbox{min} }$. Indeed, the one predicted by the DTI model is generally more elongated than the one predicted by the model that also integrates NODDI. This observation is confirmed by the fact that in all the simulations the overlapping volume between DTI and DTI-NODDI models is about 57 ± 1.4% which means that brain areas involved by the infusion differ by about 43%.

Furthermore, we can observe that the difference is not limited to the distribution shape but there is also a marked different pattern of concentration profile. This is particularly evident in the second column of Fig. 5 which displays the GD concentration on a plane perpendicular to the catheter and with an offset of 1 mm with respect to the infusion point. In this case, the DTI-NODDI model highest GD concentration is twice the one observed in the model based only on DTI. Despite this being just a qualitative example, the difference between the models in terms of concentration will find confirmation in the quantitative analysis illustrated in the next section.

Figure 5 also represents the resulting permeability vectors on different voxels as double headed arrows obtained by performing a vectorial sum of the parallel and perpendicular components of the permeability tensor and then projecting the resulting vector onto the concentration contour planes. Since the length of the arrows is proportional to permeability, the relationship between this parameter and the GD concentration profile is evident. Indeed, the GD distribution follows the main permeability directions which depend on the way the permeability tensor is defined.

### Prediction of GD Concentration

**Figure 6:**
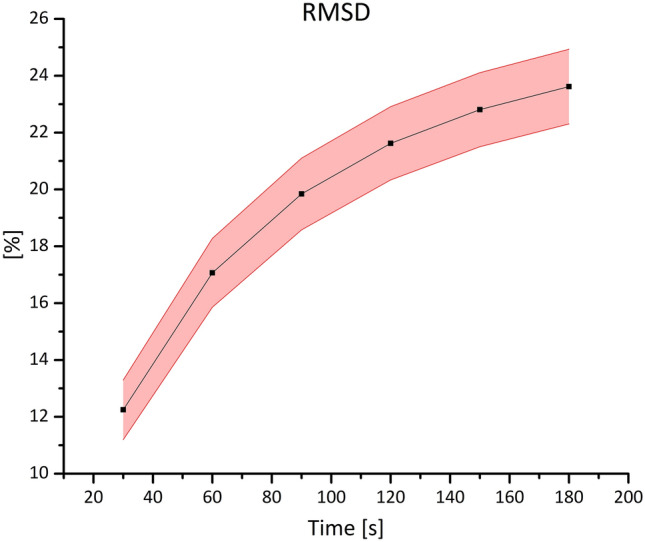
Variation of the RMSD between the two models in time (shown in terms of percentages). The figure shows the RMSD averaged between all the simulations at each time step (squared symbol). Since the data distribution at each time step is normal, the light red band indicates the standard deviation.

The analysis on drug concentration is summarized in Fig. 6 which shows the average RMSD between the simulations performed with different orientations of the catheter. This figure aims at demonstrating the difference between the models in terms of drug concentration which is fundamental in CED interventions. It is immediately possible to notice that the RMSD increases in time, going from a minimum about 12% to a maximum about 23%. Moreover, we can notice that the standard deviation is approximately constant across time even with different orientations of the catheter.

### Prediction of GD Distribution Main Direction and Infusion Penetration Length

From the PCA, we obtained the main directions along which the drug has diffused for the DTI and DTI-NODDI models for each catheter orientation. The angular difference between these directions is defined by the angle $\vartheta$. The maximum difference in terms of $\vartheta$ is around 7.92 degrees, whereas the minimum one is around 0.95. However, the average is about 4.85 degrees with a standard deviation equal to 2.54 degrees. Since the data are normally distributed, a one-sided one-sample *t*-test was used to study the results. A *p* value equal to $2.87 \times 10^{ - 4}$ demonstrates that $\vartheta$ is significantly different from zero.

**Figure 7:**
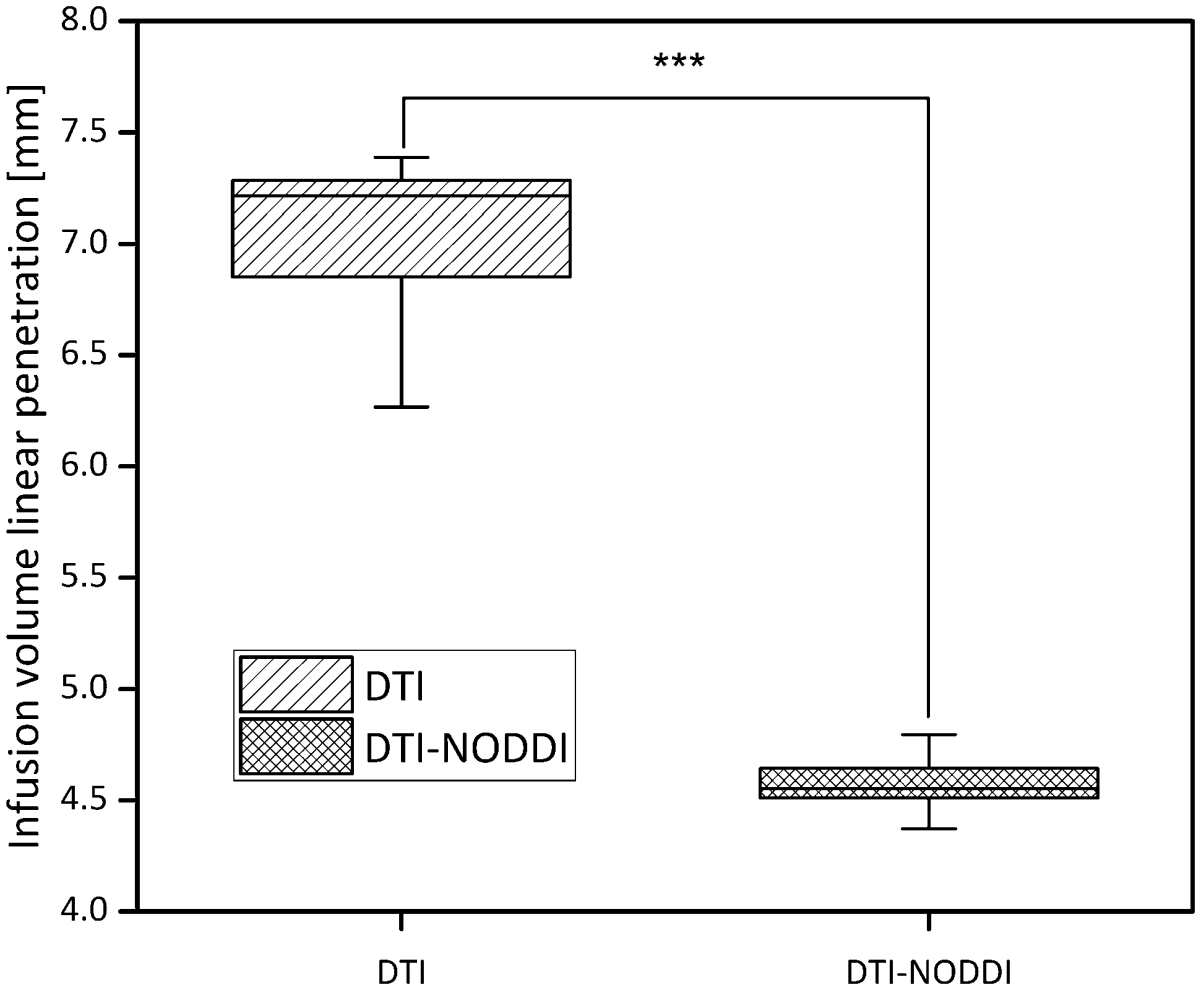
Boxplot comparing the infusion volume linear penetration length in the models. A two-sided paired *t*-test was performed to show the statistically significant difference between the models (*p* value = $5 \times 10^{ - 9}$).

Another interesting parameter to explore is the maximum linear penetration length ($L_{ \hbox{max} }$) reached by the injected volumes, to analyse how deep in the tissue the drug can distribute. This parameter was defined calculating the maximal length (at 180 s) of the infusion volumes along the direction obtained with the PCA. The $L_{ \hbox{max} }$ values for the DTI and DTI-NODDI models for each catheter orientation were then compared using a two-sided paired *t*-test. The boxplot showed in Fig. 7 brings evidence of a statistically significant difference between the two models (*p* value = $5 \times 10^{ - 9}$).

## Discussion

The proposed work comes from a relatively simple observation that was pointed out also in other studies:^28^ when injecting a drug in the brain, the final outcome in terms of drug spreading depends on the microstructural organization of the neural tissue. This is due to the fact that a drug moves in the interstitial space between glial cells, neuron cell bodies and axons, whose tortuous paths influence the main directions the infusate can take.^42^ Accordingly, the common approach adopted by many researchers is to use DTI to model this aspect. However, DTI does not consider the ${\text{VF}}_{\text{ECS}}$ which is directly related to the brain hydraulic permeability.^41^

To tackle this issue, we have developed a simplified geometrical model of the WM with the aim of defining a direct relation between the ${\text{VF}}_{\text{ECS}}$ provided by the NODDI analysis and the hydraulic permeability. The model assumes that it is possible to describe the axons arrangement as an array of parallel and rigid cylinders with the same radius. Despite this is far from being a realistic description, it succeeded in providing an analytical expression which is in very good agreement with more comprehensive studies such as^13^,^41^ (Fig. 4). Moreover, it is worth mentioning that it is very important to avoid overfitting when deriving analytical equations from numerical data. To avoid this risk, we started from two already existing equations of proven reliability^18^ and we found the best coefficients to fit our simulation results. Therefore, we can confirm the soundness of our method and combine the NODDI and DTI analysis to characterize the anisotropic permeability tensor $\varvec{K}_{{\varvec{WM}}}$.

To test the importance of including the input from NODDI in a CED predictive model, we compared our methodology with other important contributions from the state of the art,^9^,^19^,^20^ where the values of WM permeability $k_{\parallel }$ and $k_{ \bot }$, were considered constant across the brain tissue. To this end, we performed several analyses that were exhibited in the previous *Results* section.

At first, we conducted a qualitative analysis on the GD concentration contours (Fig. 5). By looking at different planes parallel and perpendicular with respect to the direction of the catheter, an important discrepancy both in terms of distribution shape and concentration is observed. Moreover, the difference concerning the overall infused volume is about 43%, meaning that there is a non-negligible impact on the brain area involved. This is particularly important in CED interventions for highly malignant brain tumors such as GBM. Since this is a dramatically aggressive tumor with a very high recurrence rate, it is crucial that the drug reaches both the most motile and the innermost cellular component of the mass, to possibly contain the spreading of the tumor. Furthermore, note that despite Fig. 5 provides an example for one simulation, these results are consistent also for all the other simulations independently from the catheter infusion directions shown in Fig. 3.

As explained in the introduction, reaching all the cells affected by a certain disease is not enough, indeed, for the treatment to be effective, it is necessary to have a concentration of drug sufficiently high. Therefore, we analyzed the models in terms of RMSD which provides a quantitative feedback in terms of concentration difference (Fig. 6). Even in this case, the catheter orientation does not play a crucial role and the RMSD increases as a function of time reaching a maximum of 23%. Such an important discrepancy implies a potentially inaccurate prediction of the outcome of the delivered therapy. Moreover, we need to take into account that, usually, CED interventions last much more than 3 min or could even be chronically implanted in patient’s brain, thus suggesting that the difference in concentration could raise even more.

Finally, we investigated the main direction that the drug takes when injected. The angle $\vartheta$ between the two models is statistically different from zero but the difference about 4.85 degrees is not impressive. This is probably due to the fact that, in both models, the eigenvectors characterizing $\varvec{K}_{{\varvec{WM}}}$ come from the same DTI dataset. Nonetheless, a $\vartheta$ value that differs significantly from zero indicates that the new definition of the permeability tensor, introduced in this manuscript, plays an important role for the overall drug distribution, which is crucial for the treatment outcome. Indeed, despite considering the same eigenvectors, the two models considered here are not equal in terms of distribution main direction, hence suggesting that knowledge of the axonal bundles principal direction is not sufficient to fully describe drug diffusion and the underlying microstructure may play a key role in this respect. A deeper parametric study, similar to those investigating the interplay between microstructure, drug distribution and infusion direction,^43^ aiming at understanding the relation between the white matter tracks orientation, the catheter placement and the drug spreading using DTI-NODDI will be the subject of further investigations.

On the other hand, comparing the infusion volume linear penetration length, we can notice a statistically significant difference (Fig. 7). The difference, which is about 3 mm, shows that the DTI model predicts a linear penetration length 50% higher than our model. Moreover, it is worth underlying that, especially in brain surgery, even a few millimetres can make the difference between a successful operation or not. Therefore, it is of paramount importance using the right numerical model.

The proposed approach has two limitations which will be tackled in future developments. The first concerns the relation between the ${\text{VF}}_{\text{ECS}}$ and the hydraulic permeability. In fact, it was derived only for the WM, where the fibres tend to be highly aligned forming bundles, because GBM tends to infiltrate and derange WM tracts. However, to have a more complete model, a relation also for the GM should be derived. Moreover, a more complex geometrical model for the WM could be used. The second limitation is given by the fact that our model needs to be validated with proper *in vivo* or *ex vivo* tests. This task, which is a very complex but fundamental step to assess the model accuracy, will be addressed in a separate contribution. Furthermore, our model would benefit from an Uncertainty Quantification analysis to understand how the variability of the model parameters impacts the predicted drug distribution.^25^ In this sense, Bayesian Inference^32^ can be used to incorporate several uncertainty sources e.g. those caused by the fact that the geometrical model cannot fully describe the WM geometry or the unavoidable experimental noise coming from the imaging acquisition process. Nonetheless, we believe that our model, which incorporates detailed information regarding the brain microstructure, offers a more comprehensive approach to brain infusion modelling that can lead to a more accurate prediction of the drug distribution.

In conclusion, in this study we have proposed an innovative model for the prediction of drug distribution in brain tissue for CED procedures. The main element of novelty comes from the idea to characterize the permeability tensor combining both DTI and NODDI images from the same subject, used as a representative case-scenario. While DTI allows distinguishing WM and GM regions and determining the orientation of the neural fibres within each voxel, NODDI provides information relative to the ${\text{VF}}_{\text{ECS}}$. By tailoring this fundamental information about the microstructure with a simplified geometrical model of the WM, we were able to assign anisotropic permeability values depending on the fibres’ directionality to each voxel. The results, analysed in terms of distribution shape, concentration profile and infusion linear penetration length, show significant differences with respect to previous DTI-based models. Specifically, the DTI model tends to overestimate the drug distribution with respect to our model. This phenomenon was detected also by Kim *et al.*^20^ by comparing their prediction with *in vivo* experiments on rat brain.

The proposed approach makes an important step further in CED modelling introducing a more comprehensive way to describe the permeability tensor. We believe that further studies, in which the brain microstructure plays a key role, could lead to a deeper understanding of the relation between modelling parameters and *non-invasive* imaging modality like NODDI. Indeed, despite detailed analyses of the neural tissue at the microscale are necessary and provides invaluable results, it is only by integrating this kind of studies with clinically feasible imaging modalities that we will be able to provide the surgeons with more effective predictive tools.

## Figures and Tables

**Figure 8:**
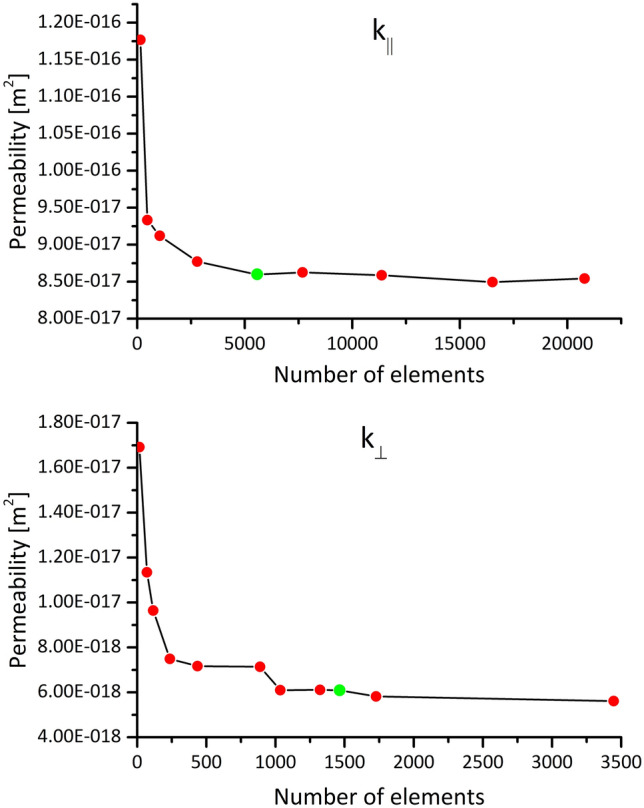
Sensitivity analysis performed on the grid of the geometrical models for both $k_{\parallel }$ and $k_{ \bot }$ with ${\text{VF}}_{\text{ECS}}$ equal to 0.15. The green marker indicates the number of elements for the mesh chosen for running all the simulations.

**Figure 9:**
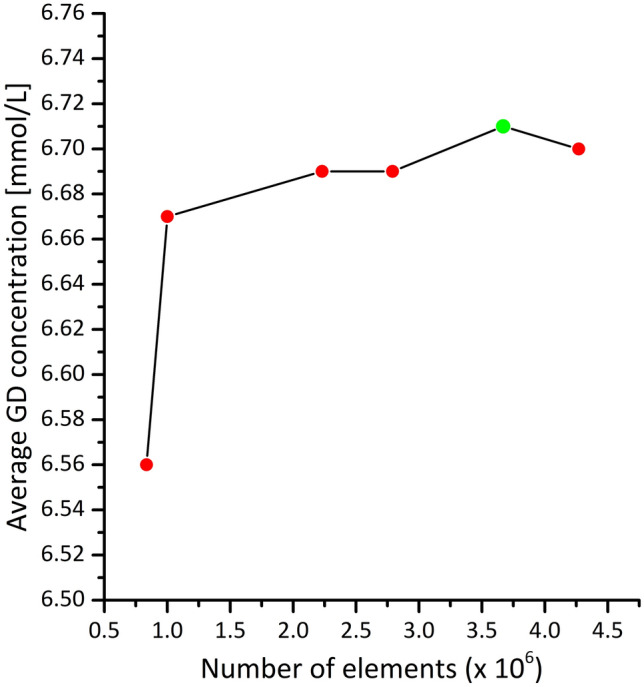
Sensitivity analysis performed on the grid of the CED model. The green marker indicates the number of elements for the mesh chosen for running all the simulations.

**Figure 10:**
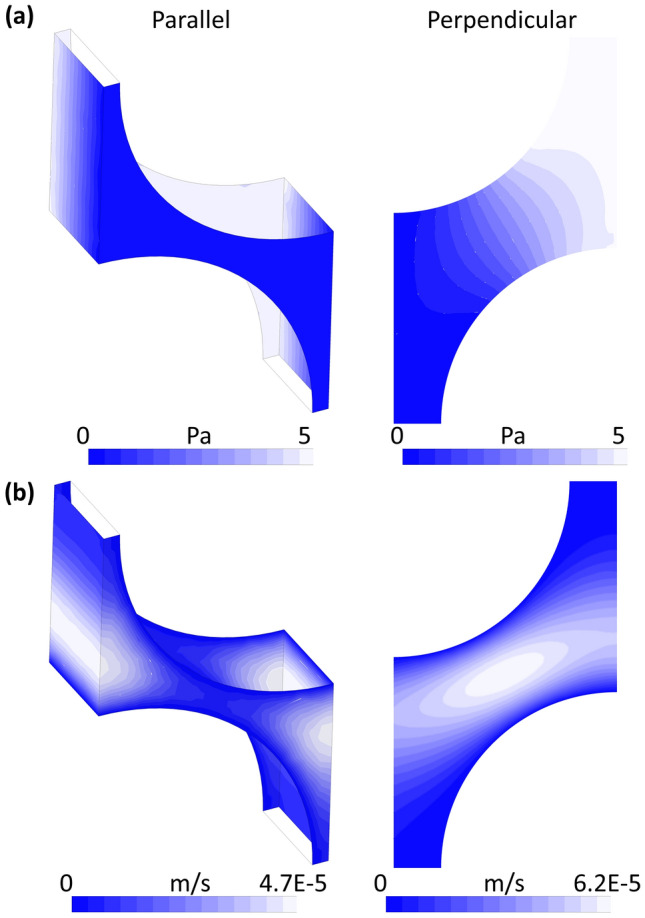
(a) Pressure contour plots computed using two RVEs along the parallel and perpendicular directions. (b) Velocity contour plots computed on the same RVEs along the parallel and perpendicular directions.

## Data Availability

The imaging dataset used in this study (Images Dataset section) is available at 10.5281/zenodo.3338449.

## Appendix 1

The grid sensitivity analysis is an important step to find a good trade-off between reducing the discretization error and the computational cost of the simulation. Accordingly, we examined several grids for each numerical model developed in this research.

First, we performed a sensitivity analysis on the geometrical models described in section *Geometrical model* whose objective was computing $k_{\parallel }$ and $k_{ \bot }$ as a function of the ${\text{VF}}_{\text{ECS}}$. For both geometries, we compared 11 grids with an increasing number of nodes achieved varying the edges discretization and the maximum face size. Since we performed the analysis on the geometries with ${\text{VF}}_{\text{ECS}}$ equal to 0.15 that are the most difficult to discretize due to the proximity of the axons, we assume that the same discretization parameter would fit also the geometries with higher ${\text{VF}}_{\text{ECS}}$. The results of the sensitivity analysis are shown in Fig. 8, the green marker indicates the number of elements for the mesh chosen for running all the simulations.

Similarly, to what has been already described, we performed a sensitivity analysis also on the CED model varying, in particular, the discretization of the catheter edges in the proximity of the inlet and the maximum dimension of the tetrahedral elements in the brain. As can be observed in Fig. 9, these simulations are intrinsically expensive because of the large number of elements. For this reason, for the sensitivity analysis only, we performed steady state simulations testing 6 different grids. We compare the simulations computing the average GD concentration in the elements comprised in a sphere (radius equal to 3 mm) centred at the catheter inlet. The sphere radius was chosen empirically to include a representative portion of the brain interested by the GD injection.

## Appendix 2

Using Darcy’s law to retrieve the relationship between permeability and ${\text{VF}}_{\text{ECS}}$, we considered two different RVEs for the parallel and perpendicular flow as explained in Section 2.4.1. In both cases, the pore length scale is comparable with the overall flow domain whereas usually the latter is much larger than the pores dimension.^40^ However, this condition is not indispensable when the spatial arrangement of the fibers is perfectly symmetric and repeatable across all the porous medium as demonstrated also in Gebart *et al*.^15^ Indeed, when considering expanded volume, which is a multiple of the RVE used in this study (independently from the chosen total length), it will be always possible to find an isobaric pressure surface at the inlet and an isobaric pressure surface at the outlet perpendicular to the flow direction (Fig. 10a). Therefore, due to the geometrical symmetry of the model, the resulting flux will be repeatable across all the medium and it will not be affected by entrance effects or unrealistic behaviors (Fig. 10b). However, it is necessary to be cautious because the assumption of a repeatable flux only holds as long as the hypothesis of having isobaric pressure surfaces, perpendicular to the flow direction both at the inlet and at the outlet of the simulation domain is satisfied.
